# Supplementary material for: Evolutionary Shift of Insect Diapause Strategy in a Warming Climate: An Intra-Population Evidence from Asian Corn Borer
Source: Biology (Basel). 2023 May 24;12(6):762. doi: 10.3390/biology12060762 (PMC10294915; doi:10.3390/biology12060762)

Figure S1: Treatment plots for field infestation.

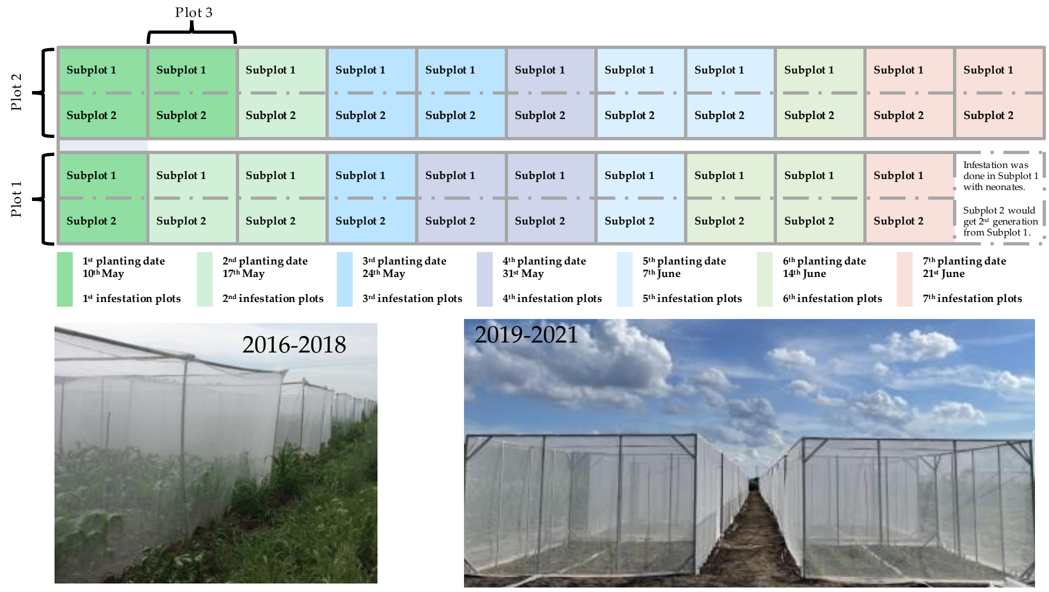

Figure S2: Dynamics of Asian corn borer moth flight in Qiqihar, Heilongjiang, China.

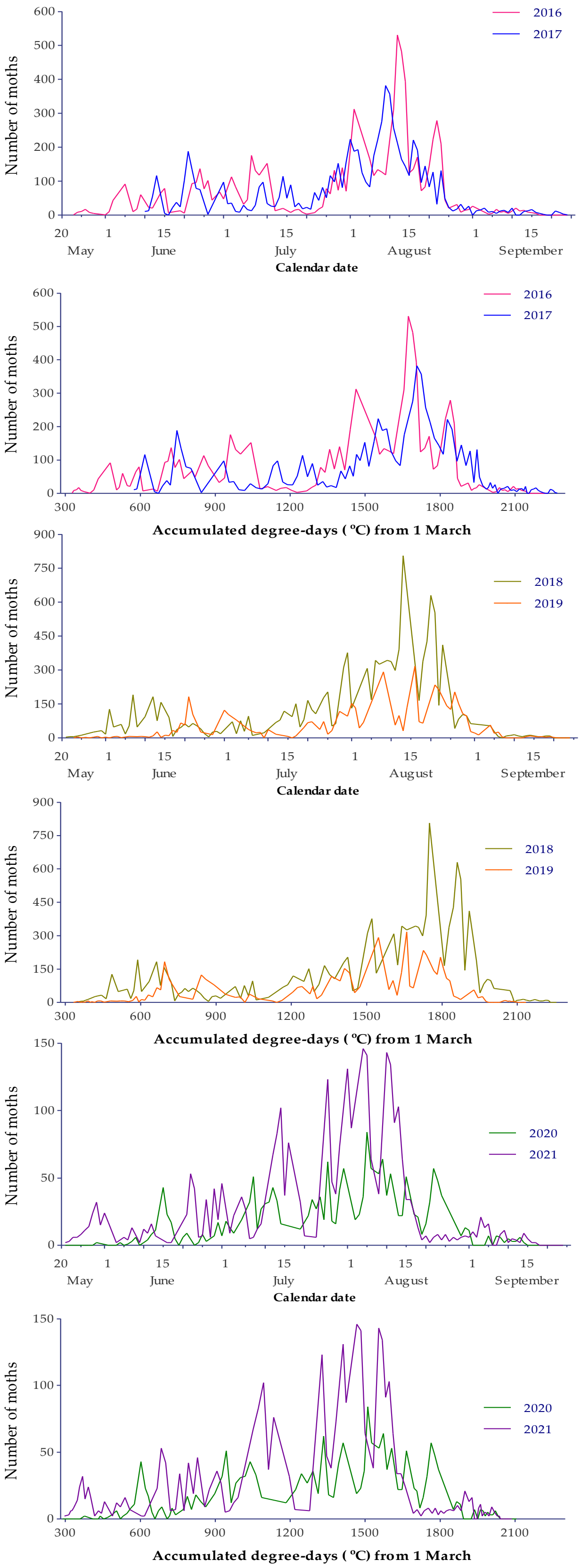

Figure S3: Proportion of the population could complete 2nd generation development.

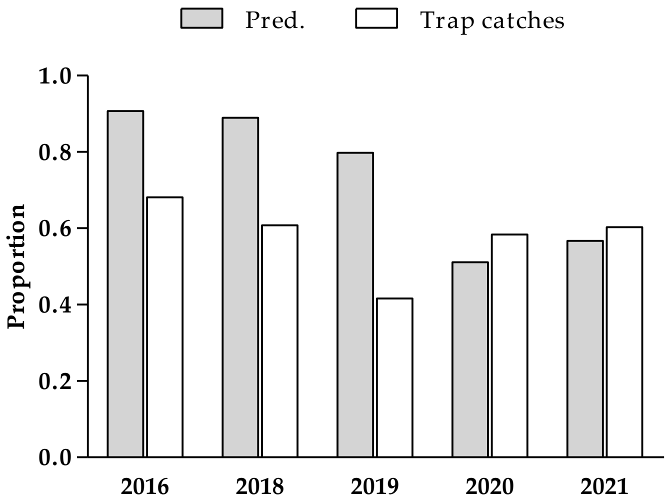

Pred.: based on model predicted bivoltine individual emergence date

Trap catches: based on light trap catch date

Difference between Pred. and Trap catches was due to adult life expectancy

Figure S4: Accumulated degree-days (°C) from 1 March in Qiqihar during 2016-2021.

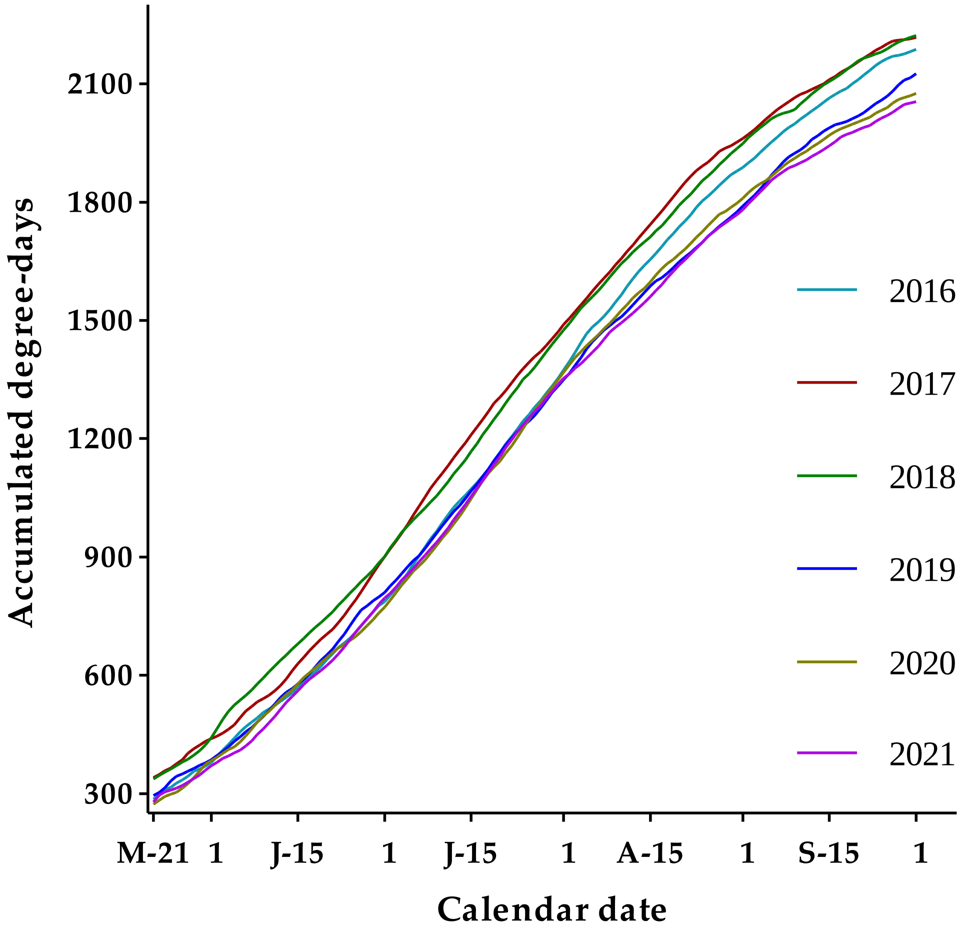

Supplement: Supplementary file 1 [file biology-12-00762-s001.zip › biology-2327950-Supplementary-Figures.pdf]
